# Correction to “Advancing FIRES Treatment: The Potential of Intrathecal Dexamethasone”

**DOI:** 10.1002/cns3.70071

**Published:** 2026-04-02

Park, C., Nordli, D.R., III, Taha, M., David, H., Kacker, S., Kass‐Hout, T., Thind, S., Hanin, A., Said, S., Haider, H.A. and Nordli, D.R., Jr. (2025). Advancing FIRES Treatment: The Potential of Intrathecal Dexamethasone. Annals of the Child Neurology Society, 3: 115–120. https://doi.org/10.1002/cns3.70008

“A Case Report and Review of the Literature” is added as the article's subtitle.

We apologize for this error.

